# Factors Influencing Leprosy Incidence: A Comprehensive Analysis of Observations in Wenshan of China, Nepal, and Other Global Epidemic Areas

**DOI:** 10.3389/fpubh.2021.666307

**Published:** 2021-05-31

**Authors:** Yu-Ye Li, Sunaula Shakya, Heng Long, Lian-Fa Shen, Yi-Qun Kuang

**Affiliations:** ^1^Department of Dermatology and Venereology, First Affiliated Hospital of Kunming Medical University, Kunming, China; ^2^NHC Key Laboratory of Drug Addiction Medicine, First Affiliated Hospital of Kunming Medical University, Kunming Medical University, Kunming, China; ^3^Wenshan Institute of Dermatology, Wenshan, China; ^4^Scientific Research Laboratory Center, First Affiliated Hospital of Kunming Medical University, Kunming, China

**Keywords:** leprosy, epidemiology, endemic, risk factors, prevalence rate

## Abstract

Leprosy remains endemic in some regions and is a global health concern. However, the possible causes and risk factors of the disease remain unclear. Data in Wenshan, China were collected from the Wenshan Institute of Dermatology (1986–2015); data in Nepal were obtained from the Leprosy Control Division, Department of Health Services, Nepal (2011 to 2015); and data from Indonesia, India, and Brazil were collected from WHO records. We assessed the epidemiological trends of leprosy in Wenshan and compared the features of possible causes and risk factors with those of other countries. We then performed a descriptive and statistical analysis to make our study more purposeful and definitive. A total of 3,376 cases were detected in Wenshan from 1986 to 2015. The overall prevalence rate (PR) of leprosy presented a decreasing trend with a peak (4.9/10,000 population) in 1986. The detection of new leprosy cases was higher in males than in females. Visible deformity increased every year since 2005 with a disability of 34.8% in 2015 among new cases. In Nepal, 2,461 leprosy patients received multi-drug therapy (MDT) in 2015 which corresponded to the PR of 0.89/10,000 population. Geographic latitude and socio-economic situations appeared to be the main causes of leprosy, and the healthcare condition was an important factor associated with leprosy incidence. The introduction of MDT effectively reduced leprosy prevalence worldwide. Wenshan (China), Nepal, and other countries share similarities in various aspects with respect to socio-cultural features, geographical distribution, environmental factors, and economic situation, which may contribute to leprosy being endemic in these areas.

## Introduction

Leprosy, also known as Hansen's disease, is an infectious disease caused by the bacillus *Mycobacterium leprae* and results in a chronic infection in humans that affects the peripheral nerves, skin, and other organs such as eyes, mucous membranes, bones, and testes (1). Transmission of leprosy is still poorly understood. Some researchers believe that leprosy is transmitted by inhalation of droplets containing the pathogenic bacteria, *Mycobacterium leprae* (*M. leprae*). However, some insist that leprosy can be transmitted via skin contact or other means. Infection of *M. leprae* results in anesthetic skin lesions, enlarged peripheral nerves, and acid-fast bacilli in the skin smear as the typical clinical signs of leprosy (2). Delayed diagnosis and treatment can lead to nerve damage presenting loss of muscle function or paralysis, even permanent disability (3). A study examined the origin and distribution of leprosy with the use of comparative genomics in 2005, indicating that leprosy most likely originated in Africa and spread to India, the New World via the slave trade, and Europe (4).

Leprosy was announced as a curable disease with the discovery of multidrug therapy (MDT). The WHO guidelines recommend a 3-drug regimen of rifampicin, dapsone and clofazimine for Multibacillary (MB) leprosy patients, and a 2-drug regimen of rifampicin and dapsone for Paucibacillary (PB) leprosy patients. (http://www.who.int/lep/mdt/regimens/en/) The implementation of leprosy elimination programs by the WHO through MDT in all the endemic countries has effectively decreased the prevalence of leprosy to <1 case per 10,000 people worldwide (5). Moreover, about 174,608 cases of leprosy were already receiving MDT worldwide in 2015, with a prevalence rate (PR) of 0.29/10,000 populations, and there has been an increment in new cases observed in the South East Asia Region from 154,834 in 2014 to 156,118 in 2015 (6). Despite being a curable disease and having had considerable success through the effectiveness of MDT, the worldwide incidence of leprosy remains high.

Although the PR of leprosy has been lower than 1/10,000 since 1982 in China (https://en.wikipedia.org), about 1,400–1,700 new cases are still being reported annually (7); accordingly, about 6,032 registered leprosy patients currently reside primarily in Yunnan, Guizhou, Sichuan, Hunan, and Guangdong provinces (http://www.lepinfo.org). The Wenshan Zhuang and Miao Autonomous Prefecture (hereinafter referred to as Wenshan), located in the southeast of Yunnan Province, is a highly endemic area for leprosy. It covers an area of 31,456 square kilometers and is further divided into eight counties, namely, Yanshan, Wenshan, Malipo, Maguan, Funing, Xichou, Guangnan, and Qiubei. The total population of the prefecture is 3.41 million (2014 estimated) and is composed of 11 ethnic groups. The terrain of Wenshan is largely composed of mountains (70%). The highest elevation in the prefecture is 2,991.2 meters above sea level and the lowest is 107 meters. It has a subtropical high plateau monsoon climate with an annual average temperature of 15.8–19.3°C and an annual rainfall of 800–1,300 mm. Leprosy was highly prevalent in the prefecture until the 1950s given the complex geographical locations and low socioeconomic development. With the decline in leprosy prevalence in this region, the morbidity was still higher than in other areas of the country until 1993 (8), despite the vertical approach implemented back then with the purpose of disease elimination (9).

Although the study of leprosy in Wenshan has been detailed for decades, it remains unclear where Wenshan is currently headed in the race to leprosy control. A previous study compared the leprosy situation in Wenshan with another place in China, and Wenshan is lagging behind in leprosy prevention and control (8). Therefore, in this study, we analyzed the trend of leprosy in Wenshan from 1986 to 2015, and compared the geographic and socio-economic status between Wenshan and four other regions where leprosy is highly endemic, aiming to identify the factors associated with leprosy endemicity and support basic scientific data for the prevention and control of leprosy in the future.

## Methods

### Ethics Statement

This study was approved by the Wenshan Institute of Dermatology and the First Affiliated Hospital of Kunming Medical University. The data analyzed in this work were anonymized.

### Study Design

The leprosy records of patients for Wenshan were maintained and collected from the Wenshan Institute of Dermatology. By the end of 2015, there were a total of 3,376 leprosy cases in Wenshan of which records were maintained. From 1986 to 2015, the detection of leprosy cases was finished through dermatologic clinics and self-reporting. There were three surveys: general survey, clue survey, and plaque valley survey that also held in case detection. The disability rate was only calculated in new cases, because most of the other cases were relapse cases; hence, we studied them separately and did not include them as previously described.

### Data Sources and Collection

The diagnosis of leprosy was based on the clinical instruction manual, histopathological features, and bacteriological index. According to the Ridley and Jopling Classification proposed in the 1960s, the MB leprosy was classified as mid-borderline (BB) leprosy, borderline lepromatous (BL) leprosy, and polar lepromatous (LL) leprosy; whereas, the PB leprosy included indeterminant (I) leprosy, polar tuberculoid (TT) leprosy, and borderline tuberculoid (BT) leprosy. Single lesion paucibacillary (SLPB) includes mainly I and TT leprosy. For treatment, a WHO criterion of clinical leprosy classification was used; in that, the presence of 1–5 lesions was classified as PB and the presence of >5 lesions was classified as MB. PB and MB patients were treated as previously described (10). Disability levels were graded according to the WHO disability grading system (11).

The data for Nepal from 2011 to 2015 was obtained from the Leprosy Control Division, Department of Health Services, Nepal. The data collected was secondary data that was approved for academic use. For further comparison, the data was obtained from previous WHO records. The data for India, Brazil, and Indonesia were also collected from WHO records. We performed a descriptive analysis of the data and attempted to identify the reasons for these regions being the top highly endemic areas of leprosy in the world.

### Statistics Analysis

All computerized data were analyzed using SPSS software. We carried out chi-square testing and logistic regression analysis to find out the delay in leprosy diagnosis and its significance in Wenshan over 30 years, and analyzed the disability rate from 1986 to 2015 in Wenshan. Then, we completed a descriptive analysis to compare the features of leprosy epidemiology and its possible causes between Wenshan and Nepal.

## Results

**Figure 1:**
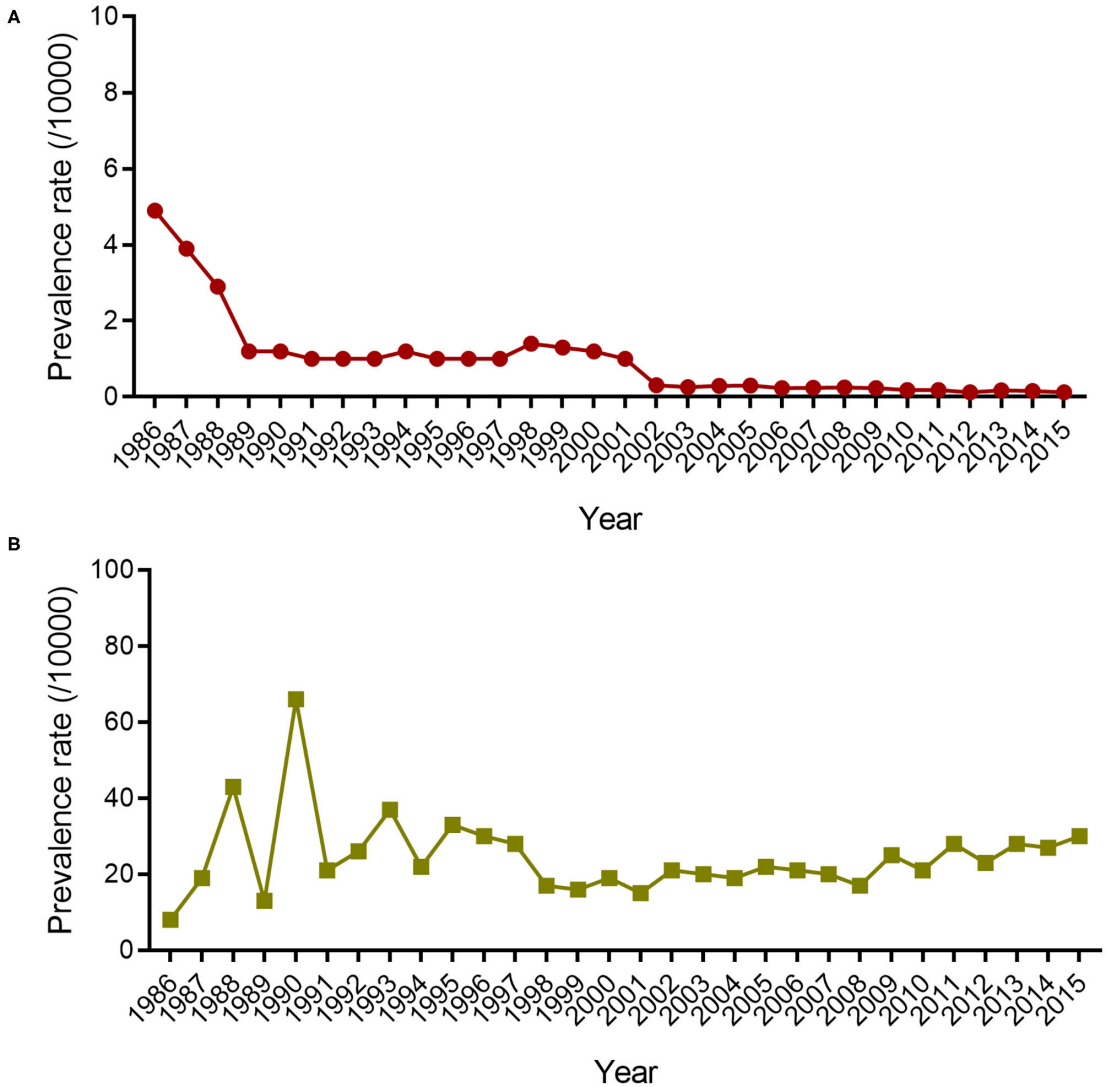
The prevalence rate and disability rate of leprosy in Wenshan. The lined dots **(A)** indicate the prevalence rate (/10,000) of leprosy in Wenshan from 1986 to 2015. The lined squares **(B)** represent the disability rate of new leprosy cases in Wenshan from 1986 to 2015.

From 1986 to 2015, a total of 3,376 leprosy cases were detected in Wenshan. The overall PR of leprosy in Wenshan showed a peak in 1986 (4.90/10,000), thereby presenting a roughly bell curve distribution between 1989 and 2015 (Figure 1). The PR in Wenshan had declined between 1986 and 1989 from 4.9/10,000 to 1.2/10,000 after the implementation of fixed-duration MDT; after that, it remained at 1.0/10,000 till 1997 based on the annual input of new patients infected before MDT (Figure 1). The PR increased and reached 1.4/10,000 in 1998 probably through nationwide active programs conducted to diagnose leprosy in China. Afterward, there was a steep decline from 1999 to 2002, and it has remained below 1/10,000 from 2001 to 2015 (0.13/10,000 in 2015). The majority of confirmed cases belonged to the Zhuang ethnic group (1,183/3,376, 35%), followed by the Miao ethnic group (864/3,376, 26%), and Han ethnic group (861/3,376, 25%); the remaining cases (468/3,376, 14%) comprised individuals from the Yi, Dai, Hui, Tu, Bai, and Zang ethnic groups. There was a higher PR of leprosy in males (2,185/3,125, 70%) than in females (940/3,125, 30%) among the new cases.

**Figure 2:**
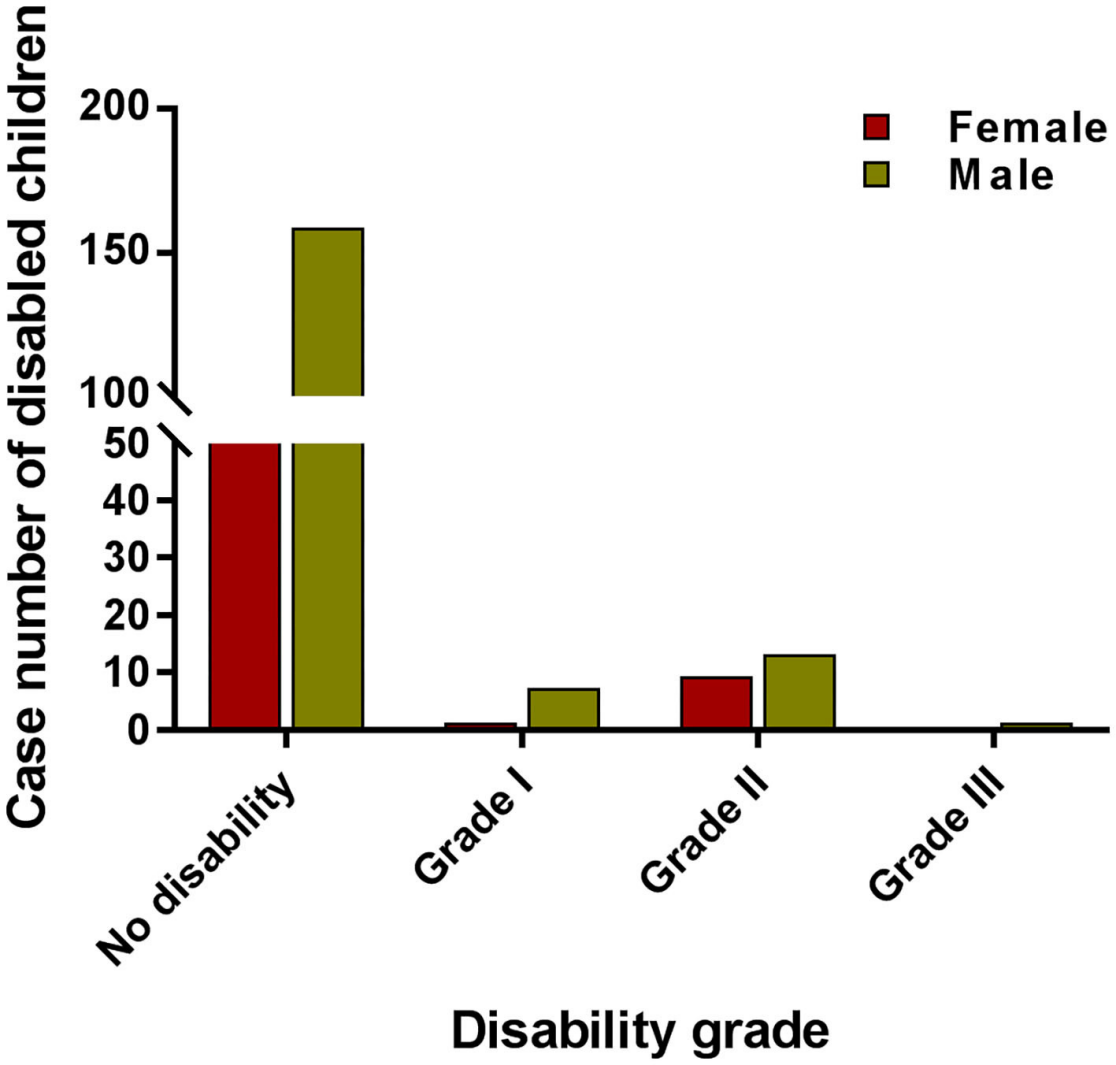
The case numbers among different grades of disabled children. The red bar indicates the case number of female children with different disability grades. The red bar indicates the case number of male children with different grade disability.

The disability rate (proportion of visible deformity) in Wenshan fluctuated and increased from 8 to 65% during 1986–1990, followed by a fluctuation (20–35%) between 1990 and 1995 (Figure 1). In the next 13 years, it had decreased to 17% until 2008, but since then it increased every year with a disability of 30% in 2015 among new cases. Among all new cases, 20.2% showed disability of Grade II and 6.4% of Grade I, and the remaining cases showed no leprosy-associated disabilities. The disability rate of Grade II was higher amongst children which called for more attention toward decreasing the ongoing transmission in the children (Figure 2).

In Wenshan, the number of MB subtype (56%) was higher than that of PB subtype (44%) for the last 30 years and it was consistent for both gender (Of MB 40% and PB 31% in male patients among all patients; MB 16% and PB 13% in female patients among all patients). The BL type was the largest subtype accounting for 38%, which was followed by BT (34%), TT (10%), BB (9%), LL (8%), and I (1%) types. The MB rate was 71.6% in 1986, after which it decreased to 64.8% in 1996 and 70% in 2015. Our study also showed that the delayed detection rate of MB leprosy was significantly higher than that of the PB (*P* = 0.003) (Table 1). The relapses tended to occur more in MB leprosy than PB in Wenshan, and delay in detection was one of the decisive causes of leprosy forms.

**Table 1:** The association of leprosy type and the delayed detection of leprosy cases in Wenshan (in year).

**Leprosy type**	**Delayed detection of case number (%)**						**χ^2^ value**	***P*-value**
	**<1 year**	**<2 years**	**<3 years**	**<4 years**	**<5 years**	**≥5 years**		
MB	768 (41.1)	579 (31.0)	268 (14.3)	106 (5.7)	55 (2.9)	92 (4.9)	17.824	0.003
PB	721 (47.8)	411 (27.3)	178 (11.8)	80 (5.3)	37 (2.5)	81 (5.4)		

**Figure 3:**
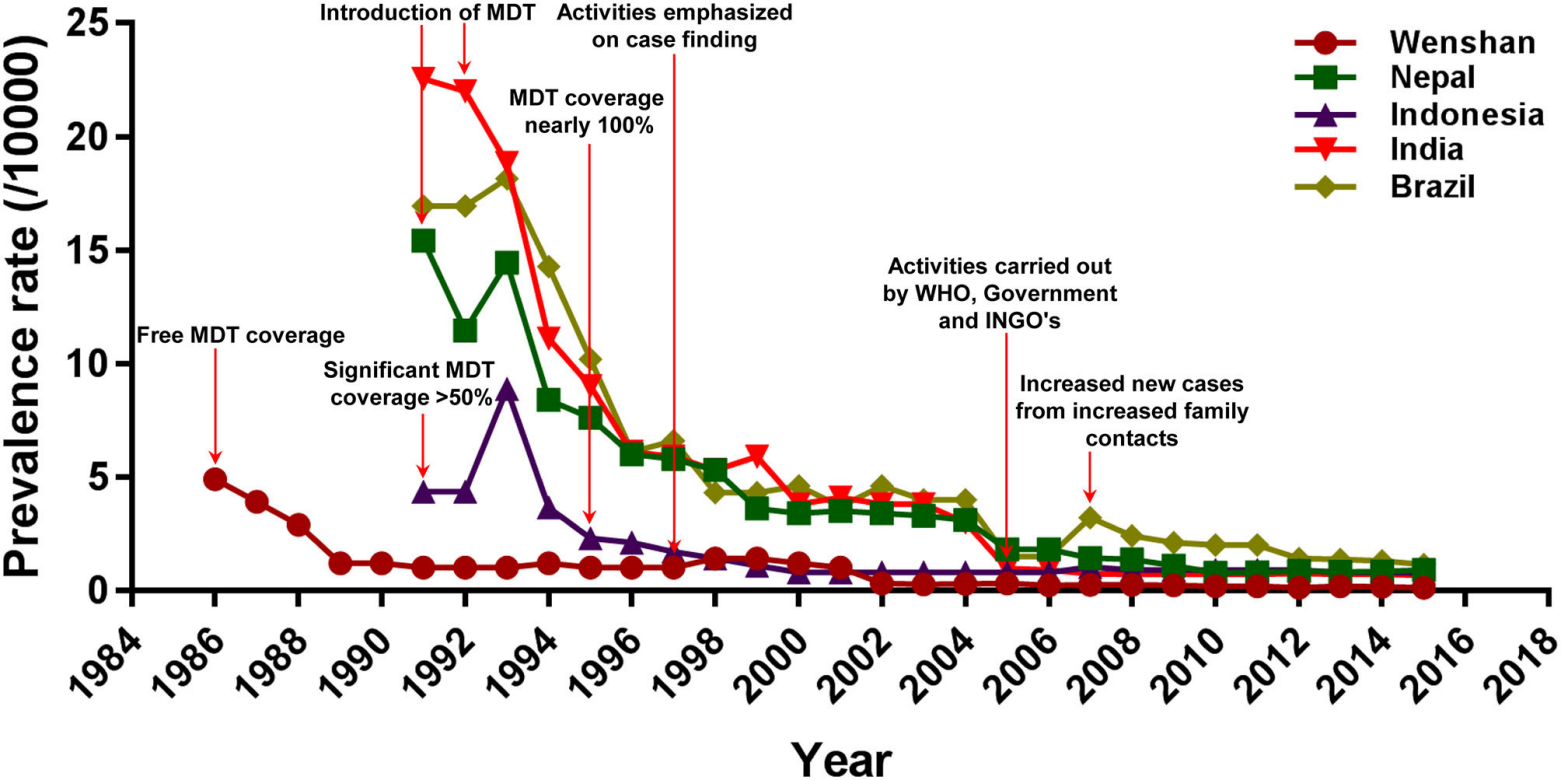
Comparison of prevalence rate and intervention strategies of leprosy between Wenshan and the other four countries. The red arrows represent the prevention and control activities conducted in different countries or regions.

Next, we compared the PR of Wenshan with that of four other high-PR countries namely Nepal, Indonesia, Brazil, and India. The overall trends of leprosy PRs in Nepal, Indonesia, India, and Brazil were similar to that seen in Wenshan (Figure 3), and the introduction of MDT obviously decreased the PR in the five countries or regions. In Nepal, there was a marked reduction in PR of registered leprosy after the implementation of MDT coverage programs as directed by WHO, and showed a fall from 15.43/10,000 in 1991 to 7.6/10,000 populations in 1995. A total of 2,461 leprosy patients were receiving MDT in 2015, which corresponded to the PR of 0.89/10,000 populations. The leprosy patients presenting to hospitals have been increasing after the elimination phase (after 2009). There was a minimal increase in PR in 2015 compared to the year before (0.83/10,000), but it has succeeded in maintaining the WHO elimination status for the last 5 years. Hence, following the continuous efforts from WHO and other international concerned agencies in cooperation with the Nepalese government, leprosy was also eliminated from Nepal at the national level in 2009 and declared in 2010.

In Indonesia, MDT was started much earlier in 1980, but the significant outcome from it was observed only in 1990 (PR: 4.34/10,000) when MDT coverage reached 52% nationwide with the help of WHO programs. There was a slight increment in the registered PR in 1993 (8.89/10,000) probably through the survey conducted during the time called “chase survey” where a follow-up of new patients was associated with health education services and voluntary skin examination of villagers. Afterward, the prevalence was markedly decreased again to 2.3/10,000 population in 1995 with 95% MDT coverage. By 2000, Indonesia had already achieved the global elimination status at the national level with a PR of 0.8/10,000 population. This can majorly be attributed to the contributions from WHO, Nippon Foundation, and Novartis providing 100% of MDT coverage throughout the country. Most recently, however, Indonesia has reported the third-highest number of new leprosy cases each year, after India and Brazil.

In India, by 1991, the PR was 22.56/10,000, which accounted for 75% of the world's leprosy cases, but the registered PR in India had already decreased to 9.0/10,000 by 1995. Elimination of leprosy was declared at the national level in India in 2005 (0.96/10,000), through a series of aggressive programs from the National Leprosy Eradication Program (NLEP). Since this announcement, funding for leprosy prevention and education programs in India has been drastically reduced. The PR remained steady from 2005 to 2015.

In Brazil, the registered prevalence of leprosy was also decreased from 16.95/10,000 in 1991 to 10.2/10,000 in 1995 highlighting the efficacy of MDT. However, Brazil still has a higher rate of transmission than the other regions or countries and thus, a higher prevalence of leprosy. Furthermore, an increased number of household contacts called known leprosy contacts (KLC) among family members is one of the reasons for a higher transmission and higher prevalence of leprosy in Brazil.

In order to determine the factors associated with leprosy and its probable modes of transmission, we compared the geographic and socio-economic situations among the top five regions or countries worldwide with high PR of leprosy. Our study showed that the ethnic minorities of the Zhuang and Miao nationalities in Wenshan were most affected, as they mostly reside in tropical and subtropical regions, while most people affected by leprosy resided in Terai, the tropical areas of Nepal. The economic development of Wenshan has been slower than other areas of the nation (GDP/capita of Wenshan was USD 2,426 which was lower than the national GDP/capita of USD 6,097). Likewise, the economic status of Nepal is also poor, with GDP per capita being USD 688 (Table 2). Hence, the negative relation between the GDP growth and the development of other social factors with the prevalence of leprosy is applicable to both these places. Nepal has low GDP, and has been supported by international institutions and WHO to the greatest extent, while this has been largely accomplished by the government of China in Wehshan. Similarly, the regions with the highest number of leprosy patients in Nepal were located in Terai, which is easily accessible by most modes of transportation. Meanwhile, the places where patients with leprosy mostly reside, such as Yanshan, Wenshan, and Qiubei are mountainous areas with difficult accessibility. Delay in case detection and unavailability of prompt referral services to general healthcare setup, along with the related low socio-economic profile, India still tends to be one of the highest leprosy endemic countries in the world.

**Table 2:** General profile comparison of risk factors of leprosy.

**Characteristic**	**Wenshan**	**Nepal**	**Indonesia**	**India**	**Brazil**
Latitude/Longitude	23°22'N-104°15'E	28°00'N-84°00'E	6°10.5'S-106°49.7'E	28°36'N-36°12'E	15°47'S-47°52'W
Area (sq.km.)	32,239	140,800	190,4569	126,9000	328,8000
Population (2014)	3.41 million	30.9 million	255.1 million	1.29 billion	204.2 million
Persons/sq.km (2014)	100	22	135	60	24.66
Climate	Subtropical	Tropical to arctic	Tropical	Tropical to montane	Tropical
Terrain	Mountains (70%)	Mountains (N) Flat plains (S)	Coastal lowlands	Plain (S) Mountains (N)	Mostly flat
Ethnic group	17 ethnicities	>100 ethnicities	300 ethnicities	6 main ethnic groups	5 major ethnic groups
GDP/capita (USD)	2,426	688	3,620	1,452	12,216
AI/capita (USD, 2013)	6,798	1,475	2,601	1,508	3,307
Unemployment rate	3.1%	2.7%	5.5%	6.6%	7.1%
Literacy rate	93% (Yunnan)	53.1%	95.38%	71.96%	92.9%

*GDP, gross domestic product; AI, annual income*.

## Discussion

Although leprosy control programs were launched very late throughout the provinces in southeast China, it had become a steady process in Wenshan Prefecture by 1986. However, there appeared to be a strong downward trend for the prevalence of leprosy in Wenshan when MDT was introduced in 1986, and the trend was continued till 1990 with a PR of 4.9/10,000 population in 1986 to 1.2/10,000 population in 1990. From 1991 to 1998, various activities for case detection were carried out such as general surveys, plaque village surveys, and clue surveys owning to the increased PR, which peaked in 1998 (1.4/10,000 population). After several leprosy elimination campaigns and health education programs that were held by the Chinese government and Yunnan Center for Disease Control and Prevention (YNCDC) from 1999 to 2002, the PR decreased to 0.31/10,000 in 2002. Meanwhile, they focused on increasing financial input for leprosy control and rehabilitation activities. Thereafter it had been slowly decreasing from 2002 till 2015 and maintained below 1/10,000 population probably through several programs such as active reporting of cases, contact surveys among family members, trace surveys, dermatology clinic reporting, and rewarding systems for reporting of new leprosy cases but less vigorously than previous activities.

In our study, we found that leprosy in Wenshan was being diagnosed more in the reproductive or child-bearing age group of male and female patients (aged 20–29 years). Meanwhile, the illiteracy rate among the affected people in Wenshan was still very high, at about 48%. About 80% of the total leprosy patients were married. From these findings, it appears that the affected patients very likely have no good idea about their disease condition and its transmission. It is probably one of the reasons for Wenshan having a higher rate of ongoing transmission and a higher number of children being diagnosed with the disease. Similarly, we also found out that the number of family contacts of leprosy cases was very high in Wenshan, with 62% of cases having positive contact history. This clearly shows that contact surveillance is not maintained properly in this region. Furthermore, the chemoprophylaxis provided to the patients has to be reviewed, as we found out that Wenshan reports a higher number of relapses and disabilities. Our data also show that the number of voluntary reporting in Wenshan has been decreasing, with only 18% of cases diagnosed this way in recent years compared to 55% cases before 1993. All these factors are likely responsible for the steady and slow control of leprosy during these recent years in Wenshan.

On the other hand, in Nepal, there was a remarkable reduction from 15.43/10,000 in 1991 to 7.6/10,000 populations in 1995 in the registered prevalence of leprosy after the implementation of MDT coverage programs by WHO. The Nippon Foundation and Sasakawa Memorial Health Foundation of Japan have provided a free supply of MDT drugs to endemic countries including Nepal during 1993–1995. Between 1995 and 2000, the Nippon Foundation defrayed the cost of drugs. Then, the pharmaceutical company Novartis AG started supplying drugs since 2000. From 2000 to 2005, Novartis' donation led to the cure of about four million patients with the provision of drugs worth USD 40 million. Hence, with the strong and sustained efforts from WHO and other international concerned agencies in cooperation with the Nepalese government, leprosy was eliminated from Nepal at the national level in 2009 and declared as such in 2010. WHO, in collaboration with its partners -the International Federation of Anti-Leprosy Associations (ILEP), the Nippon Foundation, and Novartis- developed a new strategy for the period 2006–2010 for sustaining quality leprosy control activities and thus Nepal was able to maintain its elimination status till 2015.

The Nepal Leprosy Trust, a Christian Charity founded in London, serves those affected by leprosy in Nepal. The Trust Company has established the Lalgadh Leprosy Services Centre, a hospital in Lalgadh, in southeast Nepal in 1986. Since 2000, the hospital has played a major role in carrying out community development programs that enable leprosy-affected disabled people to work together and initiate projects in their villages, such as handicrafts production for export and water and sanitation programs, among others. It also runs a Hand Surgery Camp every year, where a team of doctors from the UK stay for a fortnight and operate on all affected patients. Moreover, the Nepalese Government plans to complete a pilot prophylaxis program among long-term contacts of people recently diagnosed with leprosy. It is carried out in certain selected districts of Nepal at present but if this is proven to be successful in breaking the chain of transmission, then it will be extended nationwide. This is hoped to be a key step toward eradicating leprosy in Nepal.

Leprosy control in Wenshan has purely been an agenda carried out by the Government at the national level and has thus far been inadequate in providing sufficient rehabilitation programs to disabled patients and in controlling the current source of infection and transmission. It needs to see that people with deformity continue receiving proper treatment and care. On the contrary, the focus in Nepal has been diverted from ensuring adequate resources to enhance the gains achieved so far. Hence, the program should continue to enhance the knowledge and skills of general health service staff and ensure strong political commitment.

Leprosy has been associated with significant stigma for ages. During the medieval period, there used to be shelters where the relatively poorer people affected with leprosy were gathered together with other poor and ill subjects. These places were called leprosy asylums or leprosaria and were typically run by monks and priests; thus, such places were associated with spiritual and religious significance. People infected with leprosy were called lepers; further, it was believed that leprosy was a punishment from God and those affected were considered dead to society. Therefore, leprosy was also known as the “living death.” People affected by leprosy even had to carry a bell to warn healthy people of their arrival. Although the stigma of this disease continues, it is unclear in the modern period. Thus, investigating the risk factors for leprosy and its probable modes of transmission can help reduce the stigma associated with it, in both direct and indirect ways. The geographical and socio-cultural features of Wenshan and Nepal are similar in many ways, with both being landlocked, mountainous terrains with wide cultural diversities; therefore, we intended to study and compare the risk factors of leprosy in these areas via a descriptive approach.

Leprosy is directly related to the social factors in the population groups such as race, ethnicity, or skin color (12). De Castro et al. showed that the non-white population was more prone to leprosy (13). Previous studies have also found that environmental factors, such as soil, humidity, vegetation, and thermal-hydrologic climate also contributed as sources of leprosy transmission (14–17). In concordance with these previous studies, we found that the ethnic minorities of Zhuang and Miao in Wenshan are affected the most as they typically reside in tropical and subtropical regions, as is the case with Nepal where most people affected by the disease reside in Terai, the tropical areas (18). Hence, it can be said that people with darker skin color and residing in the tropical areas are more susceptible to leprosy. The Zhuang and Miao people also have unintelligible languages that make communication difficult with those from other regions and ethnic groups. People who practice Shigongism and Hmongism religions rely on shamans and mediums for treatment and healing. Similarly, in Nepal, people infected with leprosy mostly belong to the Brahmin who have a custom of wearing a “tika” and “sindoor” on their forehead indicating their religious beliefs or as an ornament. This tika/sindoor is made from powdered red lead and has been discussed as a possible reason to cause repeated subacute inflammation leading to a higher chance of getting infected by the leprosy bacilli (19). Similarly, the Nepalese also share a custom of eating by hands. They also believe in shamans called “dhami” and “jhakri” for healing purpose. These kinds of rudimentary customs and cultural beliefs might also be responsible for the delayed diagnosis and ongoing transmission of diseases such as leprosy in these places.

Leprosy is likely to be transmitted more in people who are involved in occupations where their skin is directly exposed to the environment and are more vulnerable to get traumatized as the wounds become lodged with *M. leprae* causing inoculation lepromas (20). The majority of people in Wenshan and Nepal are involved in agriculture as their chief occupation and most of the leprosy patients were found to be illiterate in our study. Therefore, we propose that people who indulged in occupations with higher environmental exposure such as agriculture, and those with lower education levels and lacking awareness are more susceptible to get infected with leprosy and could be one of the reasons for the higher prevalence of leprosy in both these places. This finding is consistent with previous studies (21, 22). The disease is also influenced by the socio-economic status of a person or a place and has been mentioned in previous literature as well (21–25). The economic development of Wenshan has been slower than other areas of the country, because of the difficult and remote mountainous terrain and low economic status. Moreover, Wenshan also has a high number of residents per household, poor-quality water supply, bathrooms at extreme and inconvenient places, and poor sanitation. These social factors might be contributing to an extent to the ongoing high transmission in the prefecture. Likewise, Nepal is economically underdeveloped too with a GDP per capita of USD 688, because many people still reside in remote villages that lack good water supply and other sanitary facilities. Hence, the negative relationship between GDP growth and development of other social factors with the prevalence of leprosy holds for both these places. Having said that, the pattern and mode of leprosy control in these places have been varied throughout both places where high endemic regions of leprosy exist. This could also be attributed to the geographical and economic situations in both places. Nepal with low GDP has been supported by international institutions and WHO to the greatest extent, while it has been done majorly by the government of China in Wenshan. Similarly, the regions with the highest number of leprosy patients in Nepal were located in Terai that is easily accessible by several modes of transportation. On the contrary, places like Yanshan, Wenshan, and Qiubei that house the highest numbers of people with leprosy are mountainous areas with difficult access. Therefore, these factors should also be considered for ongoing and improved leprosy control strategies in Wenshan and Nepal.

The higher prevalence of the disease in developing or underdeveloped countries can still be understood as discussed earlier. Countries like India, Brazil, Indonesia, Chad, Myanmar, and Ethiopia have higher reservoirs of infection and thereby a higher source of transmission. This can be attributed to the increased number of people per household, lack of hygiene and proper sanitation, poor and inadequate supply of water and food, lack of proper education systems and lack of awareness that is associated with the country's socio-economic development. Looking at one such country and its history of leprosy control, we thought it would be valuable to analyze the condition of leprosy in India as it contributes to 50% of the new leprosy cases worldwide (26). India is considered the point of origin of leprosy and of having spread the disease to other parts of Asia, the Middle East, North Africa, and later Europe through trade and war. India had a significant leprosy load in ancient times as the disease was chronic, contagious, and incurable at that time. By 1991, India contained 75% of the global leprosy cases (27), and the registered PR in India had already decreased from 22.56/10,000 in 1991 to 9.0/10,000 in 1995 (28). Elimination of leprosy was declared at the national level in India in 2005 (0.96/10,000) through a series of aggressive National Leprosy Eradication Program (NLEP) activities (29). Since this announcement, funding for leprosy prevention and education programs in India has been drastically reduced (30). The prevalence and rate of infection have remained steady from 2005 to 2015 (31), and there are still significant delays in treatment, both because of patients and the healthcare system and owning to a lack of knowledge about the disease. Thus, delay in case detection along with the unavailability of prompt referral services to the general health care setup (32) against the underdeveloped socio-economic profile has caused India to still be one of the highly leprosy endemic countries in the world.

Brazil is the world's eighth-largest economy by both nominal GDP and GDP as of 2017, but is still considered a developing nation. It is also one of the countries on the WHO list that has not been able to eliminate leprosy on a national level. With the efficacy of MDT, the registered prevalence of leprosy in Brazil was also decreased to 10.2/10,000 in 1995 from 16.95/10,000 in 1991 (28). However, Brazil still falls under one of the high endemic regions of leprosy in the world. Health system delays that could be contributing to the overall delayed diagnosis of leprosy due to patients who feared community isolation, those who visited a traditional healer, and those who did not think their symptoms were serious enough are the key reasons for the present high rate of transmission and thus higher prevalence of leprosy in Brazil (3). In addition, an increased number of household contacts called KLC among family members is one of the reasons for higher transmission and thus a higher prevalence of leprosy in Brazil (33).

In Indonesia, MDT was initiated much earlier in 1980, but significant outcome from it was observed only in 1990 (PR: 4.34/10,000) when the MDT coverage reached 52% nationwide with the help of WHO programs. There was a slight increment in the registered prevalence in 1993 (8.89/10,000) probably because of the survey conducted during the time called “chase survey,” where a follow-up of new patients, hand-in-hand with health education services, and a voluntary skin examination of villagers was carried out (34). Afterward, the prevalence had markedly decreased again to 2.3/10,000 population in 1995 with 95% MDT coverage. By 2000, Indonesia had already achieved the global elimination status at the national level with a prevalence of 0.8/10,000 population. This was majorly attributed to the contributions by WHO, Nippon Foundation, and Novartis providing 100% of MDT coverage throughout the country. In modern times, statistics suggest that the number of new leprosy cases in Indonesia is the third-highest number worldwide each year, after India and Brazil, with most of the cases being prevalent in Banten, West Kalimantan, South Kalimantan, and East Java (https://www.leprosymission.org).

On the contrary, economically well-developed countries like the USA, the UK, Norway, Spain, and several other European countries are still showing an increase in the registered cases of leprosy. Hence, it is important that we discuss the reasons for emerging cases of leprosy in these areas. Leprosy in Europe had declined over the years with the development of countries' socio-economic status. However, several countries in Europe such as France, Spain, and Italy have reported a number of new cases, most of the case patients are immigrants from other countries where leprosy is still prevalent (35–37). In England and Wales, 396 leprosy cases were reported between 1983 and 2012, 60% of which had South Asia as the recorded country of birth and the reporting of the cases could have been rare owing to the under-reporting done and cases gone missing (38). In Central Florida, an increased incidence of leprosy was noted since 2010, including 72 confirmed cases. Human leprosy here was linked to exposure to nine-banded armadillos and environmental sources of leprosy such as soil, plants, and water from armadillo-inhabited lands (39). Thus, as discussed, for countries with lesser-known risk factors of leprosy, eradication is still considered a challenging aim.

Globally, the number of new cases has decreased overall from 244 796 in 2009 to 202 185 in 2019. The regional proportions of all new cases in 2019 were: 71.3% (143 787) in the South-East Asian Region, 14.9% (29 936) in the Americas Region, 9.9% (20 205) in the African Region, 2.1% (4,211) in the Eastern Mediterranean Region, 1.9% (4,004) in the Western Pacific Region and 42 in the European Region. (http://www.who.int/wer) Leprosy prevalence in high endemic regions including Wenshan (China), Nepal, Brazil, and India is decreased because of the vertical system implemented. New case detection rate, on the other hand, shows to be decreasing slowly in Wenshan (from 89 in 2000 to 43 in 2015), Nepal (from 11.2 in 2011 to 11 in 2015), and Brazil, where new cases were reduced by 40,000 between 2003 to 2013 making it a reduction of 40% in 10 years (40). This is contrary to the abrupt decrease in India, wherein new cases were reduced by 420,000 from 2000 to 2006, i.e., 75% decrease in new case detection rate in only 6 years (41). In Wenshan, it can be attributed to the less vigorous amount of activities carried out post-2002 for case finding compared to 1998 and before. While in Nepal, the number of reporting of cases was increased post-2009 thereby leading to more cases being detected thereafter. In Brazil, there was significant decentralization of leprosy control activities in the country in those 10 years and also more registered cases under treatment by extended health centers (42). The disability rate in Nepal, India, and Wenshan seemed to be increasing, while it was decreasing with a reduction from 1.40/10,000 to 0.99/10,000 new leprosy cases per population during 2001–2013 in Brazil. This shows an increased delay in diagnosis in Nepal, India, and Wenshan compared to Brazil. Therefore, for countries with pocket areas of high endemicity of leprosy, concentrating on more active ways of diagnosis and treatment of current leprosy cases and maintaining the same amount of effort throughout until eradication must not be accomplished. We believe that the somewhat common backgrounds of these countries such as low socio-economic status, challenging terrain and difficult access, and education and unemployment situations play pivotal roles in the sanitation and hygiene conditions of the people residing there and are likely leading to the higher prevalence of the disease in these areas.

In our study, we have compared the prevalence rate, the intervention strategies for leprosy, and the general profile of risk factors for leprosy including latitude/longitude, terrain, and GDP in Wenshan of China, as well as in Nepal, Indonesia, India, and Brazil. However, these data were unavailable in the other four countries, the analyses of the association of leprosy type and the delayed detection of leprosy cases, the disability rate among the reported cases and children were limited to Wenshan.

## Conclusion

Our study summarizes the control and management of leprosy in Wenshan over the past 30 years. We found out that the prevalence and the new case detection rates in both places followed a declining trend. On the contrary, disability rate in the general population or among children, as well as MB rates are increasing in recent times as the ongoing rate of transmission in these areas is still high with a higher number of family contacts. Moreover, the chemoprophylaxis strategy that Wenshan has been following for treatment does not seem to be appropriately effective because of an increasing trend in the relapse rate in Wenshan. Wenshan has made remarkable efforts in the control of leprosy, but there remain sectors regarding disease management and prevention that still need to be worked upon. While highlighting the probable causes of leprosy endemism in Wenshan and Nepal, we hope it would be of some benefit to focus on these features as well.

With respect to literature from developed countries, the endemic regions of leprosy required strong commitments at both the national and international levels for the development of the country leading to proper and accessible healthcare systems, improved sanitation and hygiene, well-maintained contact surveillance, and proper and timely delivery of treatment for those in need. It would not then be impossible to control and eliminate leprosy from these regions. Additionally, the developed countries need to pay attention to the foreign-born leprosy cases. Working collectively for these measures, we can hope to progress toward eradicating this dreadful disease.

## Data Availability Statement

The original contributions presented in the study are included in the article/supplementary material, further inquiries can be directed to the corresponding authors.

## Author Contributions

Y-YL developed the idea, contributed to the study design, commented on the paper, and revised the manuscript. SS carried out the analysis and wrote the first draft. HL and L-FS carried out the analysis and commented on the paper. Y-QK contributed to the study design, explained the results, made the criticisms and suggestions, and wrote the manuscripts. All authors contributed to the article and approved the submitted version.

## Conflict of Interest

The authors declare that the research was conducted in the absence of any commercial or financial relationships that could be construed as a potential conflict of interest.
